# Plasma-Assisted Chemical Vapor Deposition of F-Doped MnO_2_ Nanostructures on Single Crystal Substrates

**DOI:** 10.3390/nano10071335

**Published:** 2020-07-08

**Authors:** Lorenzo Bigiani, Chiara Maccato, Alberto Gasparotto, Cinzia Sada, Elza Bontempi, Davide Barreca

**Affiliations:** 1 Department of Chemical Sciences, Padova University and INSTM, 35131 Padova, Italy; lorenzo.bigiani@phd.unipd.it (L.B.); alberto.gasparotto@unipd.it (A.G.); 2 Department of Physics and Astronomy, Padova University and INSTM, 35131 Padova, Italy; cinzia.sada@unipd.it; 3 Chemistry for Technologies Laboratory, Department of Mechanical and Industrial Engineering, Brescia University and INSTM, 25123 Brescia, Italy; elza.bontempi@unibs.it; 4 CNR-ICMATE and INSTM, Department of Chemical Sciences, Padova University, 35131 Padova, Italy; davide.barreca@unipd.it

**Keywords:** MnO_2_ nanostructures, plasma assisted-chemical vapor deposition, single crystal substrates, photocatalysis, magnetic materials

## Abstract

MnO_2_ nanostructures were fabricated by plasma assisted-chemical vapor deposition (PA-CVD) using a fluorinated diketonate diamine manganese complex, acting as single-source precursor for both Mn and F. The syntheses were performed from Ar/O_2_ plasmas on MgAl_2_O_4_(100), YAlO_3_(010), and Y_3_Al_5_O_12_(100) single crystals at a growth temperature of 300 °C, in order to investigate the substrate influence on material chemico-physical properties. A detailed characterization through complementary analytical techniques highlighted the formation of highly pure and oriented F-doped systems, comprising the sole *β*-MnO_2_ polymorph and exhibiting an inherent oxygen deficiency. Optical absorption spectroscopy revealed the presence of an appreciable Vis-light harvesting, of interest in view of possible photocatalytic applications in pollutant degradation and hydrogen production. The used substrates directly affected the system structural features, as well as the resulting magnetic characteristics. In particular, magnetic force microscopy (MFM) measurements, sensitive to the out-of-plane magnetization component, highlighted the formation of spin domains and long-range magnetic ordering in the developed materials, with features dependent on the system morphology. These results open the door to future engineering of the present nanostructures as possible magnetic media for integration in data storage devices.

## 1. Introduction

Manganese oxides have attracted considerable interest thanks to their diversity of oxidation states and crystal structures, yielding broadly tunable characteristics as a function of the adopted preparation conditions [1,2,3,4,5,6,7,8,9,10,11]. In particular, a remarkable attention has been focused on manganese dioxide, thanks to its low-cost, natural abundance, environmental friendliness, and versatile chemico-physical properties [12,13,14,15]. MnO_2_ exhibits at least six different structurally related crystalline modifications (*α*, *β*, *γ*, *δ*, *ε*, and *λ*) [1,2,4,7,10,16,17,18,19]. All these polymorphs are semiconductors with low resistivity [2,20,21], and have emerged as attractive candidates for several end-uses, including electrodes in Li- and Na-ion batteries and supercapacitors, thermoelectric materials, chemical sensors, photo- and electrocatalysts for pollutant degradation and hydrogen production, and magnetic devices useful for information storage [5,8,11,12,13,14,15,18,21,22,23,24,25,26,27,28,29,30,31,32,33]. Among MnO_2_ polymorphs, the most stable and abundant, i.e., rutile-type *β*-MnO_2_ (*pyrolusite*) [16,34,35], is composed of MnO_6_ octahedra linked by corner-shared oxygens into tunnel-containing frameworks [4,9,23,36]. Amid the various applications, the interest in *β*-MnO_2_ has been promoted by its screw-type magnetic structure with an important spin-lattice coupling, as well as by the large room temperature magnetoresistance and ferromagnetism. Altogether, these features are of considerable importance from both a fundamental and a technological point of view, since they can give rise to applications in recording devices and contribute to new studies on electronic-magnetic interactions in the target systems [16,24,25,35,37].

Whereas bulk manganese oxide crystals and, especially, powdered materials with different morphologies have been widely investigated [3,12,16,17,27,29,35,37,38], the fabrication and tailoring of supported thin films and nanostructures, that may yield significant changes in the system behavior, deserves further attention [6,14,15,22]. In this regard, one of the valuable means to modulate MnO_2_ nanosystem properties involves its controlled anionic doping, far less explored than the conventional cationic one. In particular, fluorine doping can be a useful tool to enhance the surface reactivity and tune both electrical and optical characteristics, a key issue for eventual photocatalytic, energy storage, and gas sensing applications [34,39,40,41,42]. In addition, the obtained system characteristics are directly affected by the electronic structure and properties of surfaces and interfaces, as well as on the nature of the used deposition substrate, which may influence the nucleation kinetics and the subsequent structural and morphological evolution [3,21]. So far, the preparation of MnO_2_ thin films/nanosystems has been performed on polycrystalline substrates by various techniques. These include reactive sputtering on Si [22], pulsed laser deposition on stainless steel [30], thermal evaporation on glass and quartz [9], hydrothermal routes on Si, carbon cloth, and Ni foams [14,33,43], electrodeposition on stainless steel, glass, carbon fibers, and Ni sheets [7,8,10,11], chemical bath deposition on stainless steel [13,30], spray pyrolysis on glass and steel [15], atomic layer deposition on Si [6,31,32], and chemical vapor deposition (CVD) on Si and glass [23,34,44]. Nevertheless, the use of single crystal substrates can not only stabilize specific polymorphs, but also affect morphology, structure, and crystal quality [21,41,42,45]. To date, different studies have reported on the atomic layer deposition (ALD) of *α*-MnO_2_ on NaCl(100), KCl(100), and KBr(100) and of *ε*-MnO_2_ on Al_2_O_3_(001) [1,21]. In addition, *λ*-MnO_2_ films have been grown on MgO(001) by plasma assisted-molecular beam epitaxy (PA-MBE) [26,27]. Films of the most stable *β*-MnO_2_ polymorph have been obtained by ALD on Al_2_O_3_(012), SiO_2_(001), and MgO(100) [1,2,21] by PA-MBE on Si(100), MgO(001), TiO_2_(110), and LaAlO_3_(001) [4,20,24,25,28], and by pulsed laser deposition (PLD) on Si(100) [5]. Nevertheless, various of these processes involved relatively harsh conditions either in terms of reaction atmosphere (e.g., use of ozone [1,2,6,21,31,32]) or of the used power/temperature [4,5,24,25,27,28,30]. In view of possible practical applications, the availability and implementation of milder and flexible preparative procedures enabling a good control over material structure, morphology, and functional properties represent an important requirement [45].

In this study, F-doped *β*-MnO_2_ nanostructures are deposited on MgAl_2_O_4_(100), YAlO_3_(010), and Y_3_Al_5_O_12_(100) single crystals, investigating the substrate influence on the resulting material chemico-physical properties. To the best of our knowledge, none of these substrates has ever been utilized so far for the growth of MnO_2_ thin films/nanostructures. For the first time, the target nanosystems are prepared by means of plasma assisted-CVD (PA-CVD), exploiting the inherent advantages and versatility of this technique for the tailored fabrication of supported materials under relatively soft operating conditions [34,45]. Mn(hfa)_2_TMEDA (Hhfa = 1,1,1,5,5,5-hexafluoro-2,4-pentanedione; TMEDA = *N*,*N*,*N′*,*N′*-tetramethylethylenediamine) [44,46], a fluorinated molecular compound, was used as a single-source precursor for both Mn and F. The obtained nanomaterials were analyzed by a multi-technique approach, involving X-ray photoelectron spectroscopy (XPS), secondary ion mass spectrometry (SIMS), field emission-scanning electron microscopy (FE-SEM), X-ray diffraction (XRD), and optical absorption measurements. In addition, the system surface morphology and magnetic characteristics were investigated by the combined use of atomic force microscopy (AFM) and magnetic force microscopy (MFM), a valuable analytical tool for the local investigation of magnetic properties [47,48,49,50].

## 2. Experimental Procedure

### 2.1. Synthesis

MnO_2_ depositions were performed using a custom-built, two-electrode plasmochemical instrumentation equipped with a radio frequency (RF) generator (*ν* = 13.56 MHz). In each experiment, one of the target single crystals (MgAl_2_O_4_(100), YAlO_3_ (010), and Y_3_Al_5_O_12_(100), CRYSTAL GmbH^®^, Berlin, 10 × 10 × 1 mm^3^, one-side polished) was fixed on the grounded electrode and used as growth substrate without any pre-treatment. The Mn(hfa)_2_TMEDA precursor powders (0.20 g for each deposition), synthesized according to the literature [44,46], were placed in an external glass vessel heated at 70 °C and transported into the deposition zone by an Ar flow (rate = 60 standard cubic centimeters per minute (sccm)). In order to avoid detrimental condensation phenomena, with consequent mass losses, the gas lines connecting the precursor reservoir and the reaction chamber were maintained at 130 °C throughout each experiment. Two separate auxiliary gas lines were used to introduce Ar (15 sccm) and O_2_ (5 sccm) directly into the reactor. Basing on previous experiments, the RF-power, total pressure, and interelectrode distance were kept constant at 20 W, 1.0 mbar, and 6 cm. Depositions were performed, for each of the three substrates, at a fixed growth temperature of 300 °C and for a duration of 90 min. Repeated growth experiments under the same conditions enabled to ascertain the full reproducibility of material chemico-physical characteristics. The use of higher growth temperatures was avoided in order to prevent MnO_2_ transformation into Mn_2_O_3_ or Mn_3_O_4_ [22,28,30,36]. For the same reason, the obtained samples were analyzed as-prepared, without any ex-situ thermal treatment.

### 2.2. Characterization

XPS analyses were performed using a Perkin–Elmer (Chanhassen, MN, USA) Φ 5600ci spectrometer, using a non-monochromatized Al Kα X-ray excitation source (*hν* = 1486.6 eV), at working pressures lower than 10^−8^ mbar. Binding energy (BE) values were corrected for charging by assigning a value of 284.8 eV to the adventitious C1s signal. Atomic percentages (at.%) were calculated by signal integration using standard Φ V5.4A sensitivity factors. Peak fitting was performed through a least-squares procedure using the XPSPEAK program [51], with Gaussian–Lorentzian sum functions. Ar^+^ sputtering was performed at 3.5 kV (Ar partial pressure = 5 × 10^−8^ mbar).

SIMS analyses were carried out at pressures lower than 1 × 10^−9^ mbar by means of a Cameca (Gennevilliers, CEDEX, France) IMS 4f spectrometer, using a Cs^+^ primary ion beam (14.5 keV, 20 nA, stability 0.1%) and negative secondary ion detection. The profiles were recorded adopting an electron gun for charge compensation, rastering over a 175 × 175 μm^2^ area and detecting secondary ions from a 8 × 8 μm^2^ sub-region in order to avoid crater effects. Signals were collected in beam blanking mode and high mass resolution configuration. Sputtering times were converted into depth using the nanodeposit thickness values measured by FE-SEM measurements.

Plane-view and cross-sectional FE-SEM analyses were carried out on a Zeiss (Oberkochen, Germany) SUPRA 40VP apparatus, at a primary beam acceleration voltage of 5.0 kV. Nanoaggregate dimensions and deposit thickness values were obtaining through the ImageJ^®^ software [52] by averaging over various independent measurements.

XRD patterns were collected by means of a Bruker (Billerica, MA, USA) D8 Advance diffractometer equipped with a Göbel mirror, using a Cu Kα X-ray source (40 kV, 40 mA). The average crystallite sizes *D* were calculated from the recorded patterns using the Scherrer formula [15,18,53]:

*D* = 0.9 [*λ*/(*FWHM* × cosθ)] (1)

where *λ* = 0.15418 nm for the Cu Kα X-ray source, whereas *FWHM* and 2θ are the peak full width at half maximum and angular position, respectively. In this work, the calculation was performed on the (101) *β*-MnO_2_ reflection [54], from which dislocation density (*δ*) and microstrain (*ε*) values were estimated through the following equations [19,34]:

*δ* = 1/*D*^2^ (2)

*ε* = *FWHM*/(4 × tgθ) (3)

Optical absorption spectra were recorded in transmittance mode at normal incidence on a Cary 50 (Agilent, Santa Clara, CA, USA) spectrophotometer, subtracting the substrate contribution in each case. Estimation of band gap (*E*_G_) values was performed through the Tauc procedure [7,8,9,30,46,48]:

(*αhν*)*^n^* = *A*(*hν* − *E*_G_) (4)

where *α* is the absorption coefficient, *hν* is the photon energy, *A* is a constant, and *n* is a coefficient dependent on the nature of the occurring electronic transitions (*n* = 2 for direct and allowed electronic transitions [15,18,23]). *E*_G_ values were obtained by extrapolating the experimental curves to intersect the horizontal energy axis at α = 0.

AFM and MFM analyses were performed using a NT-MDT (Moscow, Russia) SPM Solver P47H–PRO apparatus, operating in tapping mode and in air at atmospheric pressure. Root-mean-square (RMS) surface roughness values were obtained from 3 × 3 μm^2^ micrographs by the NT-MDT software, after plane fitting. MFM analyses were carried out using commercial cantilever tips (average height = 15 µm) coated with a CoCr magnetic layer, pre-magnetized by means of an external field. The magnetic force was measured by monitoring phase shifts in cantilever oscillations determined by tip-specimen magnetic interactions. The possible influence of electrostatic interactions was reduced by sample discharging prior to each analysis.

## 3. Results and Discussion

**Figure 1:**
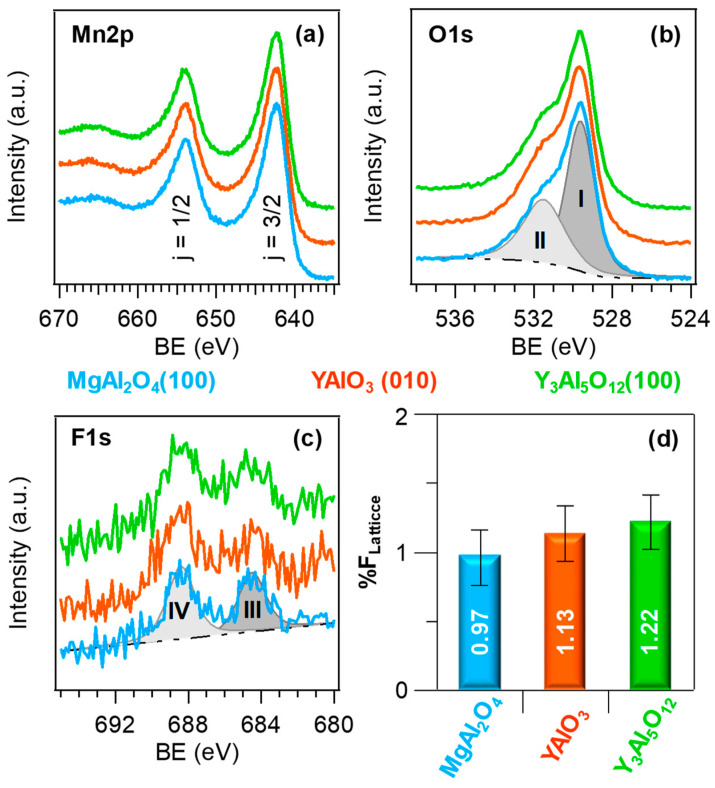
Core level Mn2p (**a**), O1s (**b**), and F1s (**c**) signals, and lattice fluorine content (**d**), for manganese dioxide systems deposited on different substrates.

The surface chemical states of the developed materials were characterized by XPS. For all the analyzed systems, only manganese, oxygen, fluorine, and carbon peaks were present in the survey scans (see Figure S1a). The disappearance of C signals upon Ar^+^ sputtering for 10 min highlighted the good system purity. In all cases, the presence of pure MnO_2_ was testified by the Mn2p signal shape and position (BE(Mn2p_3/2_) = 642.4 eV, spin-orbit separation = 11.6 eV; see Figure 1a) [4,10,14,28,44], as well as by the Mn3s multiplet splitting separation (Figure S1b). In fact, when the 3s electron is photoejected from a paramagnetic center like manganese, the exchange coupling between the 3s hole created after photoemission and the 3d electrons results in a signal splitting, whose magnitude is a fingerprint of the metal oxidation state [3,6,8,44]. In the present case, the obtained separation value was 4.7 eV, in good agreement with literature values for MnO_2_ [2,11,23,34], confirming thus the absence of other manganese oxides in the analyzed nanomaterials. The latter conclusion was further corroborated by the energy difference between the Mn2p_3/2_ and O1s lattice components (112.7 eV; see below) [23,32,34]. In fact, two components contributed to the O1s signal (Figure 1b and Figure S2a–c), a major one at 529.6 eV (I), attributed to lattice Mn–O–Mn moieties, and a second one at higher BE (II), centered at 531.5 eV, due to the presence of hydroxyl groups/oxygen chemisorbed on surface O defects [13,39,42,43]. The occurrence of the latter, already reported in previous literature studies on various manganese dioxide polymorphs [8,16,25,37], is in line with optical absorption results (see below). The surface F1s signal (Figure 1c,d, Figures S1c and S2d–f) was deconvoluted by means of two different bands, located at 684.6 eV (III) and 688.5 eV (IV). Component (III) was ascribed to lattice fluorine incorporated in manganese dioxide network, i.e., to Mn-F bonds, whereas the higher BE band (IV) located at BE = 688.5 eV was due to CF_x_ groups from precursor residuals [34,39,41,44]. Whereas band (III) was present even in the inner deposit region, band (IV) was reduced to noise level after 10 min of Ar^+^ erosion, indicating that, as already observed for carbon signals, contaminating species were limited to the system surface.

**Figure 2:**
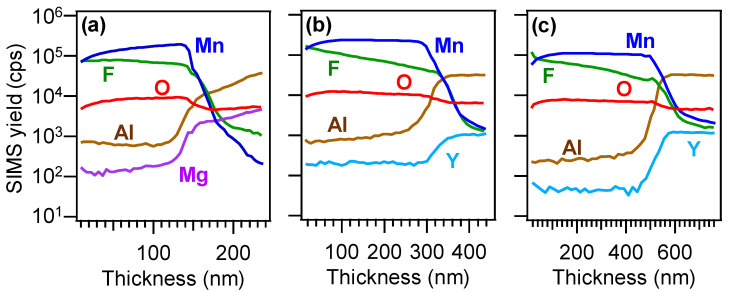
SIMS depth profiles for the specimens deposited on MgAl_2_O_4_(100) (**a**), YAlO_3_(010) (**b**), and Y_3_Al_5_O_12_(100) (**c**).

**Figure 3:**
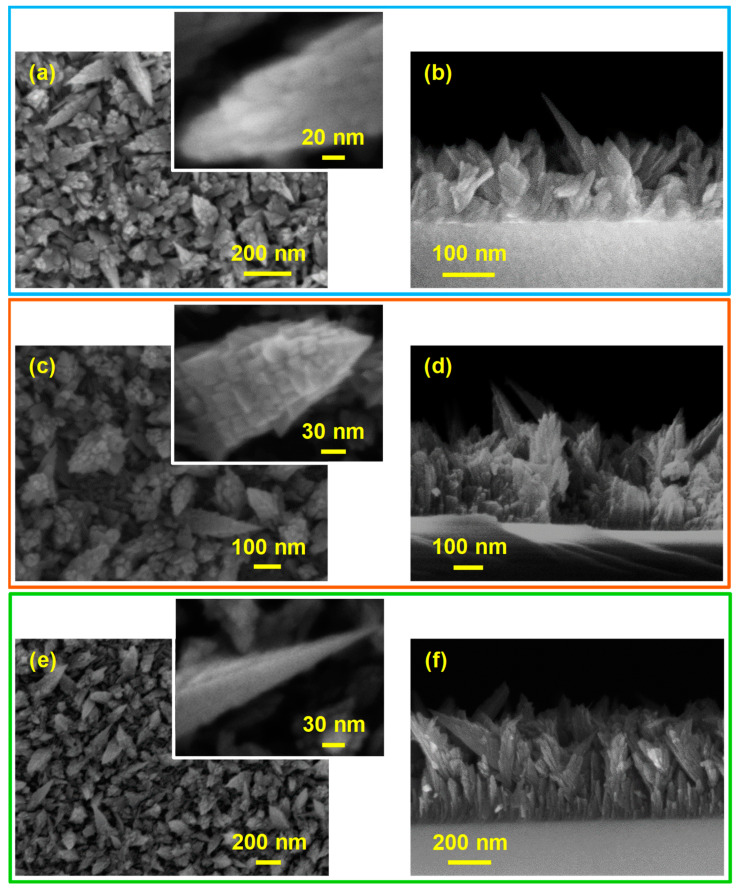
Representative plane-view (left) and cross-sectional (right) FE-SEM images for MnO_2_ nanostructures grown on MgAl_2_O_4_(100) (**a**,**b**), YAlO_3_(010) (**c**,**d**), and Y_3_Al_5_O_12_(100) (**e**,**f**).

Important information on the in-depth composition was gained by SIMS profiling (Figure 2a–c), that revealed a good material purity (mean C content lower than 10 ppm). The results highlighted an even F distribution throughout the investigated thickness, confirming a successful fluorine incorporation into manganese dioxide network. This phenomenon was traced back to the production of F• radicals deriving from precursor fragmentation in the used plasmas [34,39,40]. The almost parallel trends of manganese and oxygen signals indicated a homogeneous composition, in line with the presence of pure manganese(IV) oxide. The broadened deposit/substrate interface was related to the nano-organization of the developed systems, as revealed by FE-SEM analyses (Figure 3a–f). The recorded micrographs evidenced in fact a very open morphology, characterized by the presence of interconnected and anisotropic dendritic structures (mean width ≈ 80 nm) uniformly protruding from the underlying substrate surface. Such features might be beneficial for possible end-uses in photocatalysis [7,19,23,34,39,44], with particular regard to wastewater purification from organic pollutants and to water splitting for hydrogen production. The average length of the observed dendrites was directly affected by the used deposition substrate (220 nm, MgAl_2_O_4_(100); 200 nm, YAlO_3_(010); 270 nm, Y_3_Al_5_O_12_(010)). The observed nanoaggregates originated, in turn, from the assembly of smaller nanograins, whose dimensions, for each sample, were very close to those of the corresponding crystallites calculated by XRD analyses (see below and Figure S3). The mean deposit thickness values were estimated to be 230, 330, and 550 nm for nanomaterials supported on MgAl_2_O_4_(100), YAlO_3_(010), and Y_3_Al_5_O_12_(100). The obtainment of these different values suggested a remarkable substrate influence on precursor decomposition and nanosystem growth, all the other conditions being constant (see the Experimental section).

**Figure 4:**
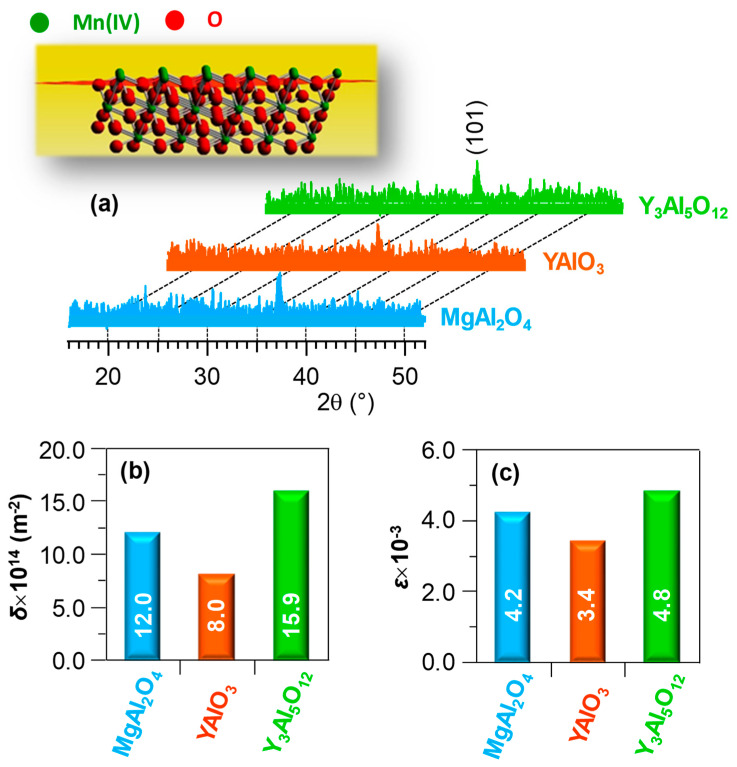
(**a**) XRD patterns of MnO_2_ specimens deposited on different substrates; inset: representation of *β*-MnO_2_ structure [54], evidencing (101) crystallographic planes. Sample dislocation density (**b**) and microstrain values (**c**).

The system structure was investigated by XRD (Figure 4a). All the recorded patterns were characterized by a single reflection located at 2θ = 37.3°, related to the (101) crystallographic planes of tetragonal *β*-MnO_2_ (*pyrolusite*; space group = *P*4_2_/*mnm*; *a* = *b* = 4.40 Å and *c* = 2.87 Å [1,4,16,21,35,37]). The presence of the sole (101) reflection irrespective of the used substrate suggested the occurrence of a (101) preferential orientation and/or of anisotropic crystallite growth [23,34,44]. The relatively weak and broad diffraction peaks, as often observed in the case of supported MnO_2_ films/nanosystems [9,10,13,14,22,33], suggested the formation of defective nanocrystallites, whose average dimensions were comprised between 25 and 35 nm (Figure S3).

The calculated dislocation density (*δ*) and microstrain (*ε*) values for the present materials (Figure 4b,c) were smaller than those reported for Si-supported MnO_2_ nanosystems [34]. In line with previous studies [19,42,55], the higher *δ* and *ε* values for the specimen supported on Y_3_Al_5_O_12_(100) corresponded to lower crystallite size dimensions (see Figure S3). This result was ascribed to the different lattice mismatch between MnO_2_ and the used substrates, highlighting the influence of the latter on the structural characteristics of the obtained systems and suggesting a lower content of dislocations and defects for materials supported on YAlO_3_.

**Figure 5:**
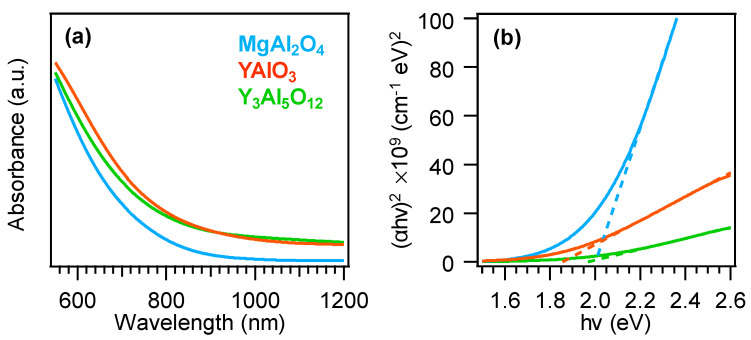
(**a**) Optical absorption spectra of MnO_2_ nanomaterials grown on different substrates and (**b**) corresponding Tauc plots.

Subsequently, attention was dedicated to the analysis of the system optical properties. All the recorded optical absorption spectra (Figure 5a) were characterized by a prominent absorption for wavelengths lower than 700 nm, corresponding to interband electronic transitions [8,23]. The broadened absorption towards the near-IR region was consistent with the presence of oxygen vacancies, as indicated by XPS analyses (see above). As a matter of fact, the occurrence of oxygen defects in the target nanomaterials can favorably influence the system functional behavior for (photo)catalytic end-uses [23,34]. In particular, the present Vis-light harvesting might be beneficial for eventual photocatalytic applications for environmental protection and energy production, as already mentioned [40,42,48]. Irrespective of the substrate nature, Tauc plot analysis (see Figure 5b) yielded a mean energy gap value of *E*_G_ = (2.0 ± 0.1) eV, which was blue-shifted with respect to that reported for various MnO_2_ polymorphs [7,9,23]. The occurrence of this phenomenon could be mainly traced back to oxygen replacement by lattice fluorine [23,41], and the almost identical band gap values were in line with the very similar fluorine contents for the present samples (see Figure 1d).

**Figure 6:**
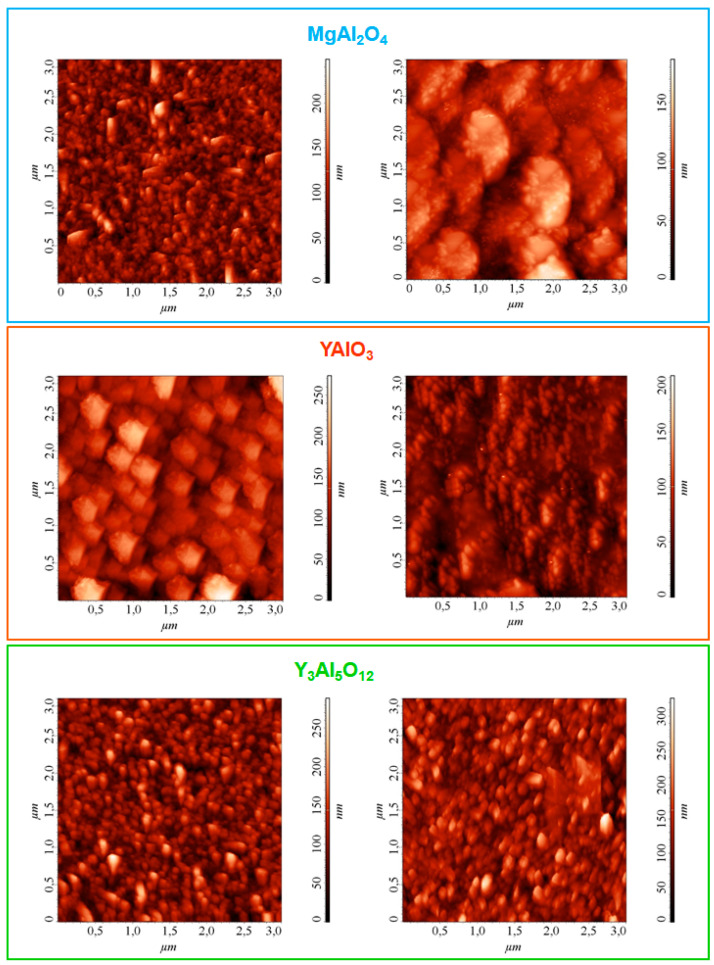
AFM (left) and MFM (right) micrographs for manganese oxide specimens deposited on MgAl_2_O_4_(100), YAlO_3_(010), and Y_3_Al_5_O_12_(100). The corresponding RMS roughness values were 28, 40, and 35 nm, respectively.

Finally, material surface topography and magnetic properties were investigated by the combined use of AFM and MFM [56,57]. AFM micrographs in Figure 6, left column evidenced a uniform interconnection of tiny aggregates for samples grown on MgAl_2_O_4_(100) and Y_3_Al_5_O_12_(100). In line with FE-SEM and XRD results (see Figure 3a–f and Figure S3), the use of YAlO_3_(010) substrate resulted in the formation of larger agglomerates and a more open morphology with a slightly higher RMS roughness, corresponding to an increased surface area [23,34]. Nonetheless, a detailed analysis of AFM images evidenced a grouping of the dendritic structures observed in FE-SEM ones, related to the tip inability to spatially resolve the single structures [34]. 

As a matter of fact, MFM analyses probe the perpendicular component of the magnetic stray field from the target systems [58]. As the magnetic tip scans over a multi-domain surface, the variations in the local magnetic stray field can attract or repel the tip, resulting thus in the contrast of the output image, which reflects the spatial distribution of magnetic domains [47,48,53]. As can be observed in Figure 6, right column, the recorded micrographs revealed an even in-plane distribution of magnetic domains. The reversing of MFM contrast from bright to dark can be associated to the switch from repulsive to attractive surface-tip interactions, corresponding, in turn, to upward and downward orientations of magnetic moments, respectively [42]. The lack of single-color large areas enabled to discard the presence of magnetic impurities in appreciable amounts, confirming thus the obtainment of pure MnO_2_ nanostructures with homogeneous characteristics.

A more detailed inspection of MFM micrographs revealed the occurrence of a multi-domain configuration directly dependent on the growth substrate. For the Y_3_Al_5_O_12_(100)-supported sample, the dimensions of magnetic domains (*D*_MFM_) and of the aggregates probed by AFM (*D*_AFM_) were comparable (*D*_MFM_ ≈ *D*_AFM_). In a different way, *D*_MFM_ was higher (lower) than *D*_AFM_ for deposits supported on MgAl_2_O_4_(100) (YAlO_3_(010)). This result indicated that, in the former case, magnetic domains were formed by different aggregates with an analogous alignment [34], while in the latter magnetic domains were separated by less abrupt walls.

Taken together, the results yielded by MFM analysis highlight the stability of the system magnetization down to the nanoscale, with tailored magnetic features and a long-range magnetic ordering. These evidences candidate the target materials for use in data storage devices. Nevertheless, the quantitative analysis of magnetic properties by the sole use of MFM is a difficult task, since the obtained magnetic signals can be overlapped with additional forces acting on the tip, such as electrostatic ones, resulting in the occurrence of topographic features in MFM images. Furthermore, as mentioned above, MFM signals are highly sensitive only to the out-of-plane magnetic stray field, preventing a straightforward prediction of a full 3D magnetic configuration [49]. Overall, these issues highlight the importance of additional analyses by complementary techniques [50] for a more detailed investigation of material magnetic properties and for further applicative research developments along this direction.

## 4. Conclusions

In summary, highly pure and oriented manganese(IV) oxide nanostructures were grown on MgAl_2_O_4_(100), YAlO_3_(010), and Y_3_Al_5_O_12_(100) single crystal substrates by PA-CVD. The obtained systems, grown under milder operating conditions with respect to various literature works, were characterized by the presence of single-phase, O-deficient *β*-MnO_2_ polymorph, the most stable and abundant one belonging to the manganese dioxide family. The use of a fluorinated molecular precursor, acting as a single-source for both Mn and F, enabled to obtain an in-situ doping of the prepared systems, with an even fluorine incorporation throughout the deposit thickness. The target materials yielded appreciable radiation absorption in the Vis spectral range, an important pre-requisite for their possible use in photocatalytic applications, such as water splitting to yield hydrogen and organic pollutant decomposition for wastewater purification. The combined use of XRD, FE-SEM, and AFM techniques evidenced that structural and morphological characteristics were directly affected by the used growth substrate. The latter also directly influenced the local variance of signals in MFM, whose utilization revealed the obtainment of spin domains with a long-range magnetic ordering, of possible interest for material application as magnetic media for integration in data storage devices. In this regard, one of the most interesting perspectives for future developments of the present work would concern a deeper investigation of the system magnetic properties as a function of fluorine content by means of complementary techniques. In addition, the outcomes yielded by this study may open up attractive perspectives for the translation of the proposed preparation route to thin films and nanosystems with a memory function for recording devices, in which reading and writing of data can be done by magneto-optical effect.

## Supplementary Materials

The following are available online at https://www.mdpi.com/2079-4991/10/7/1335/s1: Figure S1: XPS survey spectra, Mn3s signals, and total surface fluorine content for MnO_2_ samples. Figure S2: Deconvolution of surface O1s and F1s XP spectra for the target specimens. Figure S3: Mean crystallite size values for the analyzed samples.
