# Supplementary material for: Plasma-Assisted Chemical Vapor Deposition of F-Doped MnO2 Nanostructures on Single Crystal Substrates
Source: Nanomaterials (Basel). 2020 Jul 8;10(7):1335. doi: 10.3390/nano10071335 (PMC7407531; doi:10.3390/nano10071335)
Supplement: Supplementary file 1 [file nanomaterials-10-01335-s001.pdf]

nanomaterials

Supplementary Materials

Plasma-Assisted Chemical Vapor Deposition of F-Doped MnO_2 Nanostructures on Single Crystal Substrates

Lorenzo Bigiani [1], Chiara Maccato [1,*], Alberto Gasparotto [1], Cinzia Sada [2], Elza Bontempi [3] and Davide Barreca [4]

1 Department of Chemical Sciences, Padova University and INSTM, 35131 Padova, Italy; lorenzo.bigiani@phd.unipd.it (L.B.); alberto.gasparotto@unipd.it (A.G.)
2 Department of Physics and Astronomy, Padova University and INSTM, 35131 Padova, Italy; cinzia.sada@unipd.it
3 Chemistry for Technologies Laboratory, Department of Mechanical and Industrial Engineering, Brescia University and INSTM, 25123 Brescia, Italy; elza.bontempi@unibs.it
4 CNR-ICMATE and INSTM, Department of Chemical Sciences, Padova University, 35131 Padova, Italy; davide.barreca@unipd.it
* Correspondence: chiara.maccato@unipd.it; Tel.: +39-0498275234

Received: 3 June 2020; Accepted: 4 July 2020; Published: date

S1. Chemico-physical Characterization

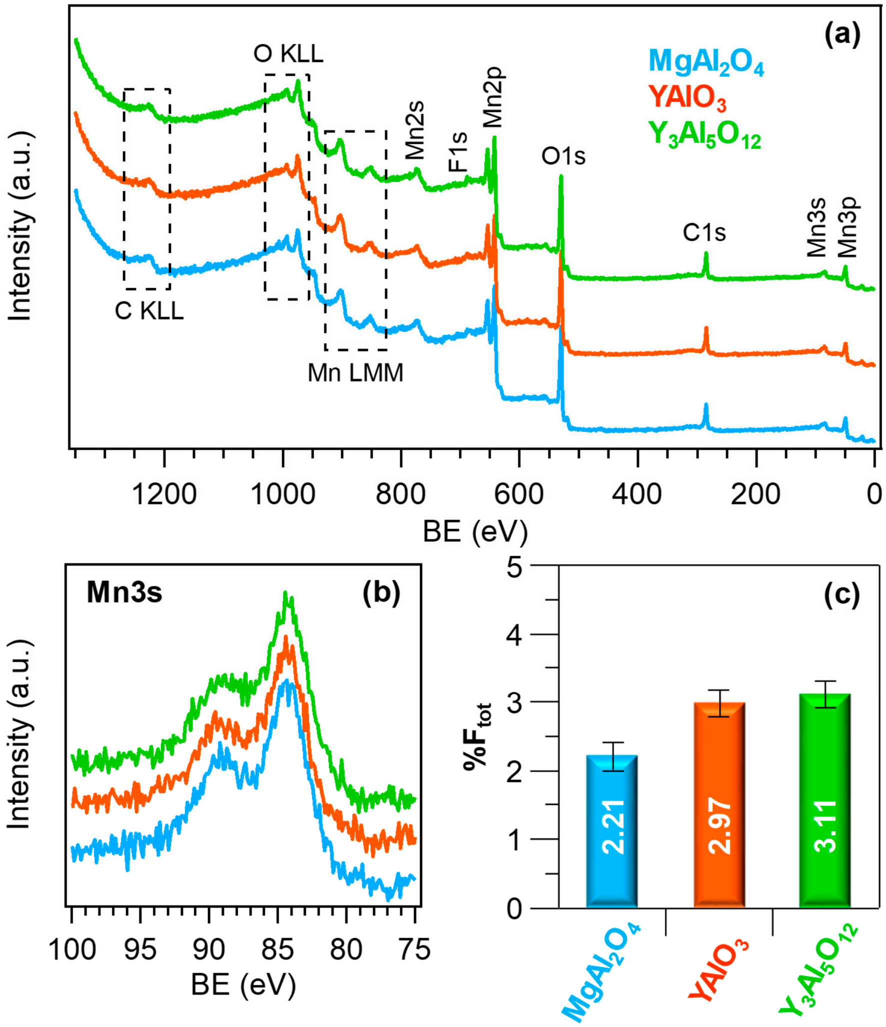

Figure S1 (**a**) Wide-scan XP surveys for MnO_2 samples deposited on $MgAl_2O_4$(100), $YAlO_3$(010) and $Y_3Al_5O_{12}$(100). (**b**) Mn3s photopeaks and (**c**) total surface fluorine content for the different analyzed specimens.

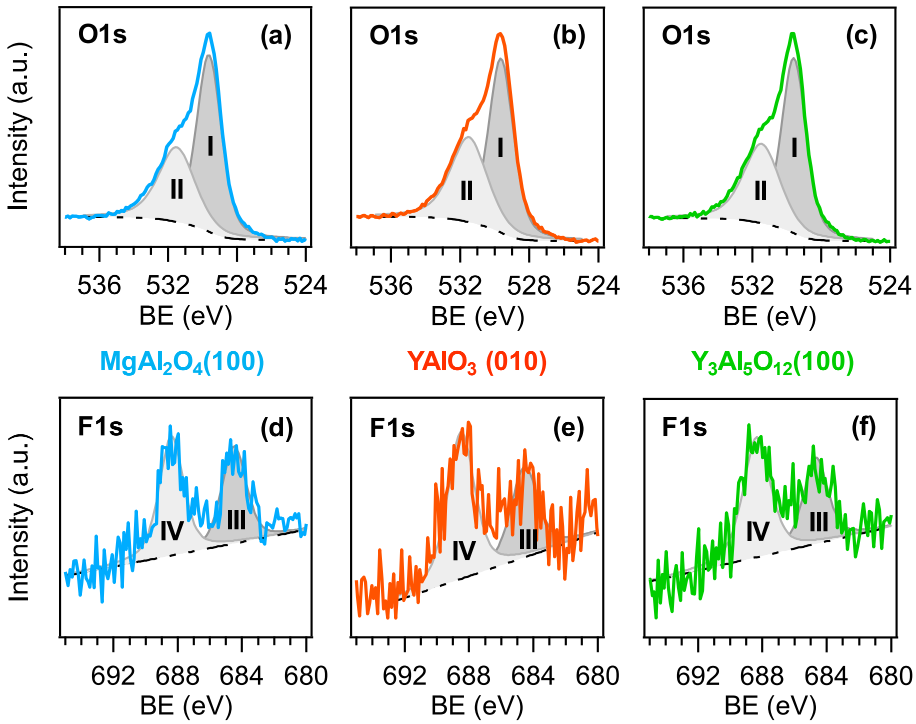

Figure S2. Surface O1s (**a–c**) and F1s (**d–f**) spectra, along with the corresponding fitting components, for MnO_2 specimens. The contribution of component (II) to the total O content was estimated to be 39.0%, 43.0% and 41.0% for samples supported on $MgAl_2O_4$(100), $YAlO_3$(010) and $Y_3Al_5O_{12}$(100), respectively. The contribution of component (IV) to the total F content was estimated to be 56.0%, 62.0% and 61.0% for samples supported on $MgAl_2O_4$(100), $YAlO_3$(010) and $Y_3Al_5O_{12}$(100), respectively.

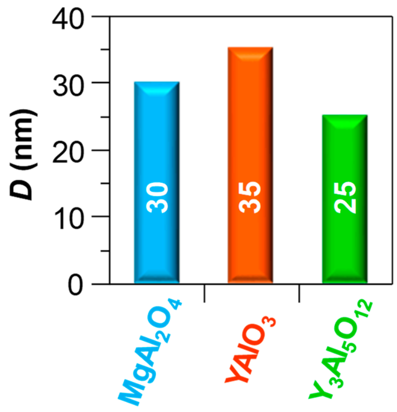

Figure S3. Crystallite size (*D*) values for MnO_2 samples deposited on different substrates.
